# Matrix stiffness promotes glioma cell stemness by activating BCL9L/Wnt/β-catenin signaling

**DOI:** 10.18632/aging.202449

**Published:** 2021-02-01

**Authors:** Bei Tao, Yi Song, Yao Wu, Xiaobo Yang, Tangming Peng, Lilei Peng, Kaiguo Xia, Xiangguo Xia, Ligang Chen, Chuanhong Zhong

**Affiliations:** 1 Department of Rheumatology and Immunology, Affiliated Hospital of Southwest Medical University, Luzhou, China; 2 Department of Neurosurgery, Chongqing University Three Gorges Hospital, Chongqing, China; 3 Sichuan Clinic Research Center for Neurosurgery, Luzhou, China; 4 Department of Neurosurgery, Affiliated Hospital of Southwest Medical University, Luzhou, China

**Keywords:** matrix stiffness, glioma, stemness, Wnt/β-catenin

## Abstract

Matrix stiffness is a key physical characteristic of the tumor microenvironment and correlates tightly with tumor progression. Here, we explored the association between matrix stiffness and glioma development. Using atomic force microscopy, we observed higher matrix stiffness in highly malignant glioma tissues than in low-grade/innocent tissues. *In vitro* and *in vivo* analyses revealed that culturing glioma cells on stiff polyacrylamide hydrogels enhanced their proliferation, tumorigenesis and CD133 expression. Greater matrix stiffness could obviously up-regulated the expression of BCL9L, thereby promoting the activation of Wnt/β-catenin signaling and ultimately increasing the stemness of glioma cells. Inhibiting Wnt/β-catenin signaling using gigantol consistently improved the anticancer effects of chemotherapy and radiotherapy in mice with subcutaneous glioma tumors. These findings demonstrate that a stiffer matrix increases the stemness of glioma cells by activating BCL9L/Wnt/β-catenin signaling. Moreover, we have provided a potential strategy for clinical glioma treatment by demonstrating that gigantol can improve the effectiveness of traditional chemotherapy/radiotherapy by suppressing Wnt/β-catenin signaling.

## INTRODUCTION

Glioma is one of the most common brain-associated malignant tumor, and has a high risk of invasion and recurrence [1, 2]. Standard treatments (surgery combined with chemotherapy or radiation) have greatly improved the outcomes of glioma patients in recent years [3]. However, many glioma patients still have a poor prognosis due to aggressive tumor growth and invasion [1–3], and even convalescent patients are at high risk for tumor recurrence due to residual cancer stem cells (CSCs) [4–7]. Thus, there is an urgent need to elucidate the underlying mechanisms of glioma development, and to design novel treatments to improve the anticancer effects of glioma therapy.

CSCs are a small subpopulation of cells in tumor tissues with the capacity for self-renewal, differentiation and enhanced tumorigenicity, and their presence is strongly associated with tumor initiation and development. The proliferation and maintenance of CSCs depend on a variety of factors, including the expression of stem-associated transcription factors or the activation of pro-survival signals by tumor cells, the secretion of cytokines by stromal cells, and the characteristics of the extracellular matrix [4, 7, 8]. Carcinoma tissues display mechanical heterogeneity in terms of their stiffness [9], and recent findings have indicated that the stiffness of the extracellular matrix activates the stemness of several tumor types, including lung cancer and colorectal cancer [10–13]. Glioma stem cells tend to be distributed in invasive front tissues, which have a stiffer matrix than core glioma tissues [12, 14–17]. Thus, different matrix stiffness levels could directly induce different tumor phenotypes.

Wang and colleagues reported that biomechanical force signals from compounds in the extracellular matrix induced pro-survival pathways such as AKT/SRY-box 2 signaling and Yes-associated protein 1-induced Nestin activation, ultimately conferring a stem-like phenotype on cancer cells [18]. However, it is unclear whether and how the matrix stiffness influences the stemness of glioma cells and the risk of tumor progression. In the present study, we assessed the matrix stiffness of glioma tissues with different degrees of malignancy. We also cultured glioma cells on tunable polyacrylamide hydrogels to determine the effects of the matrix stiffness on glioma cell stemness and *in vivo* tumorigenesis. Then, we determined the signaling pathway through which the matrix stiffness altered the stemness of glioma cells, and evaluated the effects of inhibiting this pathway in combination with chemotherapy/radiotherapy. Our results have revealed an innovative approach for clinical glioma treatment.

## RESULTS

### Greater matrix stiffness increases the stemness of glioma cells

**Figure 1:**
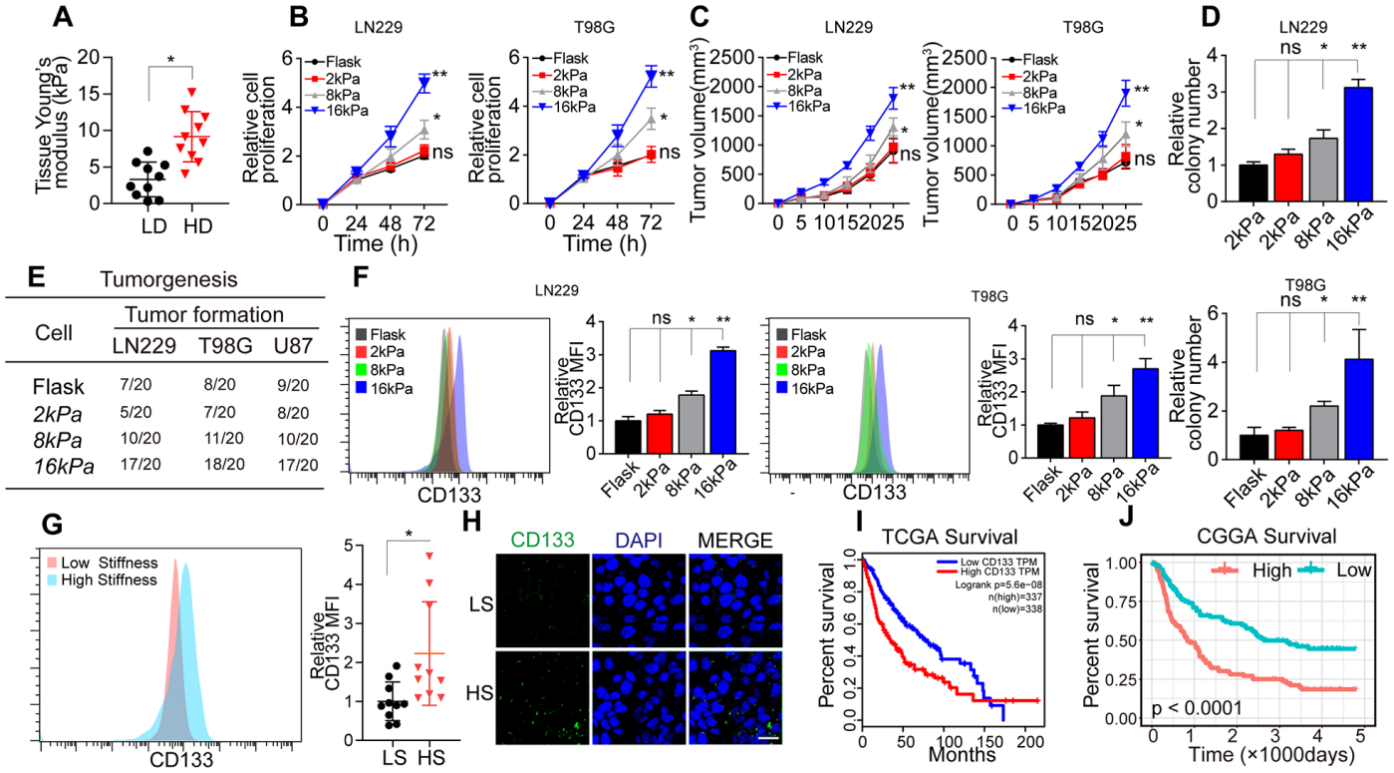
**Greater matrix stiffness promoted stemness in glioma.** (**A**) Tissue stiffness of tumors derived from the high-degree/malignant (HD) and low-degree/innocent (LD) groups. (**B**) The relative proliferation of LN229 or T98G cells pre-cultured on gels of different stiffness levels was detected at various time points. (**C**) Tumor volumes of mice injected with LN229 or T98G cells pre-cultured on gels of different stiffness levels. (**D**) The relative number of cell colonies formed by LN229 or T98G cells pre-cultured on gels of different stiffness levels was detected at various time points. (**E**) Tumorigenesis of mice subcutaneously injected with 10^4^ LN229 or T98G cells pre-cultured on gels of different stiffness levels. (**F**) Flow cytometry analysis of CD133 expression in LN229 or T98G cells cultured on gels of different stiffness levels. (**G**) Flow cytometry analysis of CD133 expression in tissues derived from the HS and LS groups. (**H**) Immunofluorescence analysis of CD133 expression in tissues derived from the HS and LS groups. Scale bar, 50 μm. (**I**) Kaplan-Meier analysis of *CD133* mRNA expression in patients from The Cancer Genome Atlas database (N=675). (**J**) Kaplan-Meier analysis of *CD133* mRNA expression in patients from the Chinese Glioma Genome Atlas database (N=325). *P < 0.05, **P < 0.01, n.s. no significant difference.

To assess the correlation between matrix stiffness and tumor progression in glioma, we collected tumor tissues from glioma patients and divided them into the high-degree/malignant group and the low-degree/innocent group. A tissue stiffness analysis using atomic force microscopy indicated that high-degree/malignant tumor tissues had a stiffer matrix (average 8.6 kPa) than low-degree/innocent tumor tissues (average 3.8 kPa) (Figure 1A). These results suggested that greater matrix stiffness promotes the progression of glioma.

To further investigate the effects of the matrix stiffness on glioma cells, we seeded LN229 and T98G glioma cells on tunable polyacrylamide hydrogels with stiffness levels of 2 kPa (soft), 8 kPa (middle stiffness) and 16 kPa (stiff). The glioma cells seeded on 16 kPa gels exhibited an enhanced proliferative phenotype *in vitro* (Figure 1B) and *in vivo* (Figure 1C). A colony formation assay (Figure 1D) and tumorigenesis analysis (Figure 1E) also indicated that the tumorigenesis of glioma cells was enhanced when the cells were cultured on 16-kPa gels.

CSCs promote tumorigenesis and tumor development, and enhanced stemness may increase tumor cell proliferation and tumor formation [5–7]. Thus, we speculated that a stiffer matrix might promote a proliferative phenotype and tumorigenesis by enhancing the stemness of glioma cells. As anticipated, the expression of the glioma stem cell marker CD133 was clearly upregulated in the stiff cell culture group (Figure 1F), indicating that a stiffer matrix increases tumor stemness. Consistently, CD133 expression was greater in higher stiffness (HS) glioma tissues than in lower stiffness (LS) glioma tissues (Figure 1G). To confirm our results, we also analyzed glioma tissues from The Cancer Genome Atlas (Figure 1I) and the Chinese Glioma Genome Atlas (Figure 1J), and found that greater *CD133* expression was associated with poorer survival in glioma patients. These results suggested that a stiffer matrix enhances the stemness of glioma cells, thus promoting tumorigenesis, sustained glioma tumor growth and poorer patient outcomes.

### Greater matrix stiffness promotes glioma stemness by inducing BCL9L

**Figure 2:**
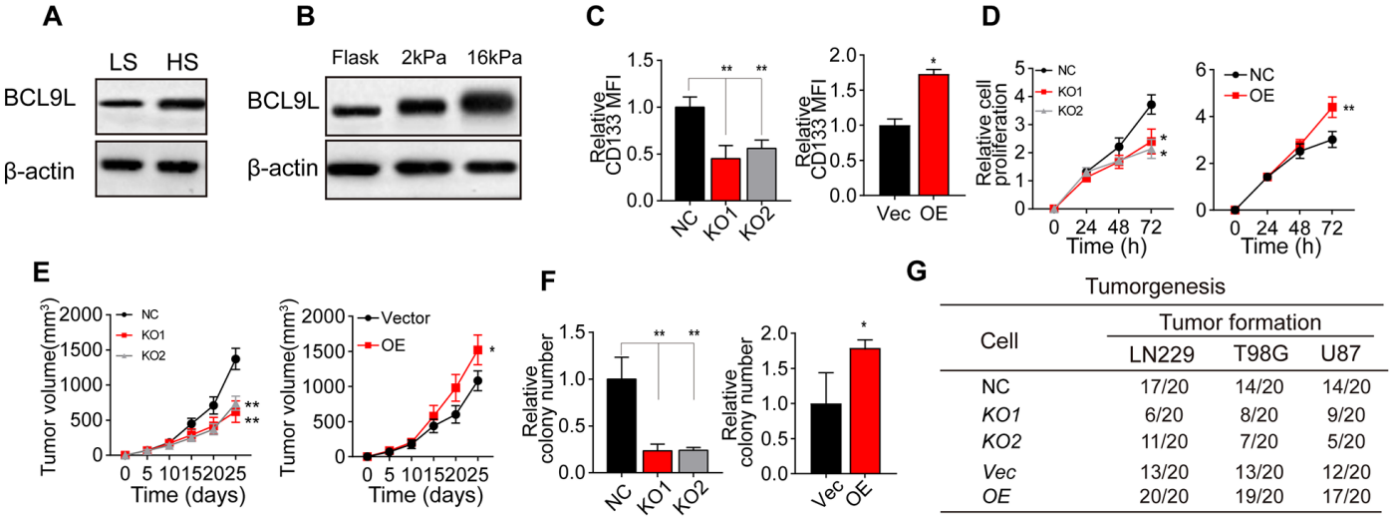
**Greater matrix stiffness increased glioma stemness by inducing BCL9L.** (**A**) Western blotting analysis of BCL9L expression in tissues derived from the HS and LS groups. (**B**) Western blotting analysis of BCL9L expression in LN229 cells cultured on gels of different stiffness levels. (**C**) Flow cytometry analysis of CD133 expression in LN229 cells treated with negative control shRNA (LN229-NC), *BCL9L* shRNA #1 (LN229-KO1) or *BCL9L* shRNA #2 (LN229-KO2) and cultured on 16-kPa stiffness gels, and in LN229 cells treated with the control vector (LN229-Vec) or *BCL9L* overexpression vector (LN229-BCL9LOE) and cultured on flask dishes. (**D**) The proliferation of LN229-NC, LN229-KO1, LN229-KO2, LN229-Vec or LN229-BCL9LOE cells pre-cultured on 16-kPa stiffness gels, and of LN229-Vec or LN229-BCL9LOE cells cultured on flask dishes. (**E**) Tumor volumes were measured at various time points in mice injected with LN229-NC, LN229-KO1 or LN229-KO2 cells pre-cultured on 16-kPa stiffness gels, and in mice injected with LN229-Vec or LN229-BCL9LOE cells cultured on flask dishes. (**F**) The cell colonies formed by LN229-NC, LN229-KO1 or LN229-KO2 cells pre-cultured on 16-kPa stiffness gels and by LN229-Vec or LN229-BCL9LOE cells cultured on flask dishes were detected at various time points. (**G**) Tumorigenesis of mice subcutaneously injected with 10^4^ LN229-NC, LN229-KO1 or LN229-KO2 cells pre-cultured on 16-kPa stiffness gels, or injected with LN229-Vec or LN229-BCL9LOE cells cultured on flask dishes. *P < 0.05, **P < 0.01, n.s. no significant difference.

It has been well documented that Wnt/β-catenin signaling is a critical determinant of CSC pluripotency and self-renewal [19–24]. BCL9 and BCL9L are transcriptional co-activators within the Wnt enhanceosome [25–32]. We performed Western blotting to compare BCL9L levels between HS and LS glioma tissues, and found that BCL9L was upregulated in HS tissues (Figure 2A). Consistently, BCL9L expression was greater in glioma cells cultured on 16-kPa gels than in those cultured on soft stiffness gels or flask (Figure 2B and Supplementary Figure 1A).

To confirm the involvement of BCL9L in matrix stiffness-induced tumor stemness, we knocked down *BCL9L* in LN229/T98G cells using short hairpin RNA (shRNA) technology (Supplementary Figure 1B) and overexpressed *BCL9L* in LN229/T98G cells (Supplementary Figure 1C). Intriguingly, knocking down *BCL9L* suppressed the upregulation of CD133 in glioma cells cultured on 16-kPa gels, whereas overexpressing *BCL9L* further enhanced CD133 expression (Figure 2C and Supplementary Figure 1D). In addition, silencing *BCL9L* impaired glioma cell proliferation, whereas overexpressing *BCL9L* strengthened it, both *in vitro* (Figure 2D and Supplementary Figure 1E) and *in vivo* (Figure 2E and Supplementary Figure 1F). Similar effects were observed in colony formation and tumorigenesis assays (Figure 2F, 2G, Supplementary Figure 1G), even under high-stiffness culture conditions. These results indicated that BCL9L is a crucial promoter of matrix stiffness-induced tumor stemness.

### Greater matrix stiffness induces glioma stemness by activating BCL9L/Wnt/β-catenin signaling

**Figure 3:**
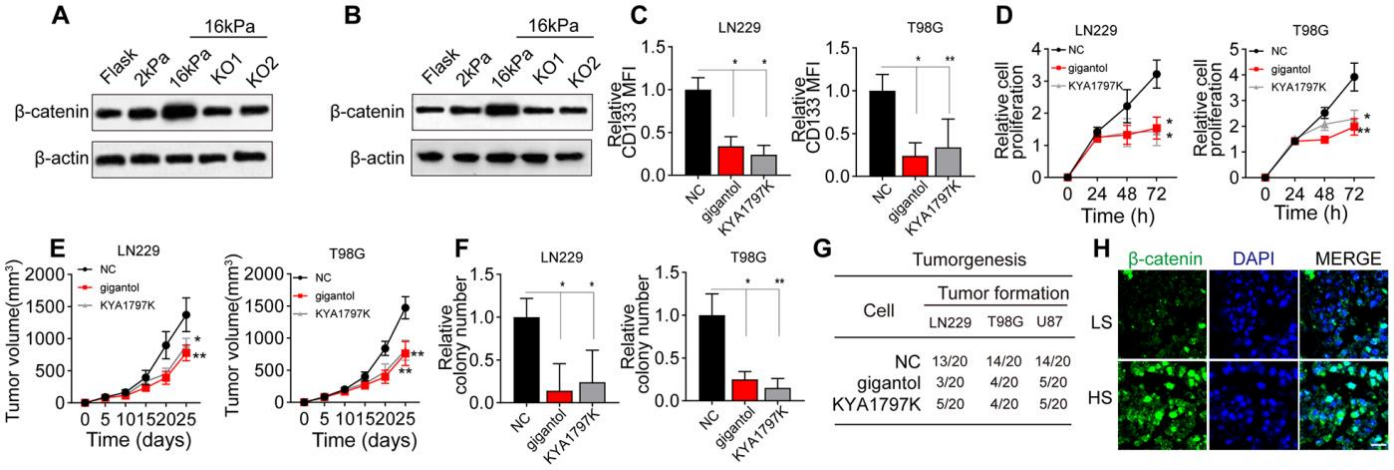
**Wnt/β-catenin signaling was activated in glioma cells cultured on high-stiffness gels.** (**A**) Western blotting analysis of β-catenin expression in LN229 cells cultured on gels of different stiffness levels with or without *BCL9L* shRNA treatment. (**B**) Western blotting analysis of β-catenin expression in T98G cells cultured on gels of different stiffness levels with or without *BCL9L* shRNA treatment. (**C**) Flow cytometry analysis of CD133 expression in LN229 or T98G cells cultured on 16-kPa stiffness gels and treated with PBS, gigantol (100 μM, 72 h) or KYA1797K (25 μM, 72 h). (**D**) Proliferation of LN229 or T98G cells treated with PBS, gigantol (100 μM, 72 h) or KYA1797K (25 μM, 72 h) and cultured on 16-kPa stiffness gels. (**E**) Tumor volumes were measured at various time points in mice injected with LN229 or T98G cells that had been treated with PBS, gigantol (100 μM, 72 h) or KYA1797K (25 μM, 72 h) and cultured on 16-kPa stiffness gels. (**F**) Cell colony formation of LN229 or T98G cells treated with PBS, gigantol (100 μM, 72 h) or KYA1797K (25 μM, 72 h) and cultured on 16-kPa stiffness gels. (**G**) Tumorigenesis of mice subcutaneously injected with 10^4^ LN229 or T98G cells that had been pre-treated with PBS, gigantol (100 μM, 72 h) or KYA1797K (25 μM, 72 h) and cultured on 16-kPa stiffness gels. (**H**) Immunofluorescence analysis of β-catenin expression in tissues derived from the HS and LS groups. Scale bar, 50 μm. *P < 0.05, **P < 0.01, n.s. no significant difference.

Next, we detected the effects of the matrix stiffness on Wnt/β-catenin signaling downstream of BCL9L in glioma cells. Notably, Wnt/β-catenin expression was greater in the stiff group than in the soft and middle-stiffness groups, but the silencing of *BCL9L* reversed this phenomenon (Figure 3A, 3B). To confirm the involvement of Wnt/β-catenin signaling in glioma, we treated glioma cells with gigantol and KYA1797K, two inhibitors of Wnt/β-catenin. As anticipated, gigantol or KYA1797K treatment significantly suppressed the upregulation of CD133 in glioma cells cultured on 16-kPa gels (Figure 3C). The inhibition of Wnt/β-catenin also remarkably suppressed the proliferation of glioma cells *in vitro* (Figure 3D) and *in vivo* (Figure 3E), and weakened their colony formation (Figure 3F) and tumorigenesis (Figure 3G). Consistently, when we compared Wnt/β-catenin signaling between HS and LS glioma tissues, we found that β-catenin expression was elevated in the HS group (Figure 3H). These results indicated that greater matrix stiffness promotes glioma stemness by activating Wnt/β-catenin signaling downstream of BCL9L.

### Inhibiting Wnt/β-catenin signaling strengthens the anticancer effects of glioma treatments

**Figure 4:**
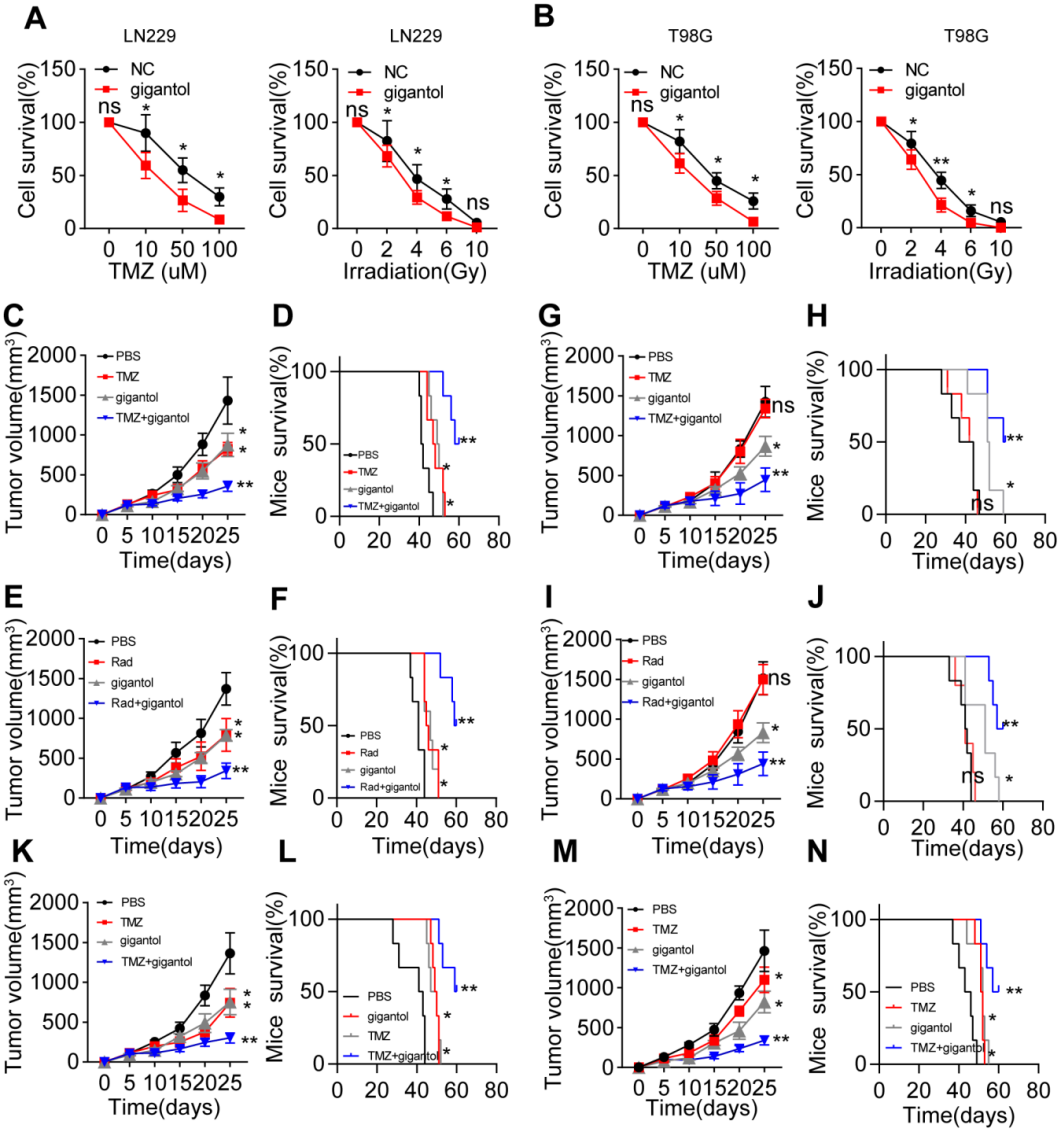
**Suppressing Wnt/β-catenin signaling enhanced the anticancer effects of chemotherapy and radiotherapy.** (**A**) Left: Survival of LN229 cells treated with different concentrations of temozolomide (TMZ; 0, 10, 50 or 100 mM) combined with PBS or gigantol (100 μM) on 16-kPa stiffness gels for 48 hours. Right: Survival of LN229 cells treated with different doses of radiotherapy (0, 2, 4, 6 or 10 Gy) combined with PBS or gigantol (100 μM) on 16-kPa stiffness gels. (**B**) Left: Survival of T98G cells treated with different concentrations of TMZ (0, 10, 50 or 100 mM) combined with PBS or gigantol on 16-kPa stiffness gels for 48 hours. Right: Survival of T98G cells treated with different doses of radiotherapy (0, 2, 4, 6 or 10 Gy) combined with PBS or gigantol (100 μM) on 16-kPa stiffness gels. (**C**, **D**) Tumor volumes (**C**) and survival (**D**) were analyzed at various time points in LN229-bearing mice treated with PBS, TMZ (10 mg/kg), gigantol (10 mg/kg) or TMZ (10 mg/kg) combined with gigantol (10 mg/kg). (**E**, **F**) Tumor volumes (**E**) and survival (**F**) were analyzed at various time points in LN229-bearing mice treated with PBS, radiotherapy (10 Gy), gigantol (10 mg/kg) or radiotherapy (10 Gy) combined with gigantol (10 mg/kg). (**G**, **H**) Tumor volumes (**G**) and survival (**H**) were analyzed at various time points in mice injected with LN229 cells that had been cultured on 16-kPa stiffness gels and treated with PBS, TMZ (10 mg/kg), gigantol (10 mg/kg) or TMZ (10 mg/kg) combined with gigantol (10 mg/kg). (**I**, **J**) Tumor volumes (**I**) and survival (**J**) were analyzed at various time points in mice injected with LN229 cells that had been cultured on 16-kPa stiffness gels and treated with PBS, radiotherapy (10 Gy), gigantol (10 mg/kg) or radiotherapy (10 Gy) combined with gigantol (10 mg/kg). (**K**, **L**) Tumor volumes (**K**) and survival (**L**) were analyzed at various time points in T98G-bearing mice treated with PBS, TMZ (10 mg/kg), gigantol (10 mg/kg) or TMZ (10 mg/kg) combined with gigantol (10 mg/kg). (**M**, **N**) Tumor volumes (**M**) and survival (**N**) were analyzed at various time points in U87-bearing mice treated with PBS, TMZ (10 mg/kg), gigantol (10 mg/kg) or TMZ (10 mg/kg) combined with gigantol (10 mg/kg). *P < 0.05, **P < 0.01, n.s. no significant difference.

Given that Wnt/β-catenin signaling is crucial for maintaining glioma stemness, we wondered whether blocking Wnt/β-catenin signaling could improve the anticancer effects of glioma treatments [21]. The presence of CSCs correlates closely with sustained tumor growth, which is usually accompanied by the development of resistance to chemotherapy or radiotherapy [19]. To determine whether inhibiting Wnt/β-catenin signaling could improve the efficacy of traditional clinical interventions for glioma, we treated LN229/T98G cells with gigantol in combination with the chemotherapeutic agent temozolomide or radiotherapy. Gigantol treatment significantly increased the cytotoxicity of temozolomide treatment and radiotherapy to LN229 (Figure 4A) and T98G cells (Figure 4B).

To confirm the anticancer effects of gigantol, we subcutaneously injected mice with LN229 cells and treated them with phosphate-buffered saline (PBS), gigantol, temozolomide, radiotherapy or gigantol combined with temozolomide/radiotherapy. As anticipated, the suppression of Wnt/β-catenin signaling with gigantol remarkably enhanced the tumor suppression and prolonged the overall survival of mice treated with chemotherapy (Figure 4C, 4D) or radiotherapy (Figure 4E, 4F). To simulate highly malignant glioma patients, we also established a subcutaneous tumor mouse model using LN229 cells cultured on 16-kPa gels, in which we previously observed that β-catenin expression was elevated. Intriguingly, in this model, gigantol treatment alone exhibited obvious anticancer effects, whereas chemotherapy or radiotherapy alone only exhibited slight anticancer effects, possibly indicating that treatment resistance occurred due to the activation of Wnt/β-catenin signaling (Figure 4G–4J). Importantly, the combination of gigantol with chemotherapy also significantly suppressed the tumor development and prolonged the survival of mice injected with T98G or U87 cells (Figure 4K–4N). These results indicated that the suppression of Wnt/β-catenin signaling could improve the efficacy of chemotherapy or radiotherapy in glioma treatment.

## DISCUSSION

In this study, we assessed the effects of the matrix stiffness on the tumor stemness of glioma cells. Our results demonstrated that a stiffer matrix increased the stemness of glioma cells, thus enhancing their proliferation and tumorigenesis. We found that a stiffer matrix activated BCL9L and its downstream signals Wnt/β-catenin, whereas inhibiting Wnt/β-catenin signaling improved the anticancer effects of traditional clinical interventions, revealing a potential approach to glioma treatment (Supplementary Figure 2).

CSCs, also known as tumor-initiating cells or tumor-repopulating cells, are a rare cell subpopulation, and their presence is strongly associated with cancer initiation and development [5–7, 19, 20]. Although much research has been conducted on CSCs, the nature of these cells remains controversial [6]. There is increasing evidence that CSCs are plastic and that extracellular matrix-like conditions (e.g., 3D matrix culture systems) can facilitate the development of stem cell-like properties and promote the tumorigenesis of cancer cells [8, 10, 12, 14, 33–39]. Moreover, compelling research has demonstrated that compounds such as collagen and fibronectin from the extracellular matrix can directly stimulate pro-survival signaling pathways and promote stemness in tumor cells [33, 38]. A stiffer extracellular matrix was reported to increase the stemness of liver cancer cells [40]. Additionally, glioma and hepatoma CSCs were found to be less proliferative in a softer matrix [17, 39]. However, another report indicated that cancer cells with high levels of stem-like markers were more likely to enter dormancy and reduce their proliferation in stiff gels, while they tended to initiate tumorigenesis in a soft tissue matrix [41]. Our results indicated that tumor cells cultured on soft gels (6 kPa) presented poor proliferative characteristics, despite their low apoptosis rate. On the other hand, a stiffer matrix promoted the development of a stem-like phenotype, characterized by increased levels of CD133-positive CSCs and enhanced tumorigenesis and tumor growth. These findings supported the concept of plastic CSCs, and prompted us to further explore the correlation of the matrix stiffness with glioma stem cell remodeling.

In terms of the mechanism, one important finding of this study was that BCL9L was upregulated under stiff matrix conditions, suggesting that BCL9L could be a novel biological marker of CSCs. Previous studies have indicated that the Wnt/β-catenin signaling pathway activates the pluripotency and self-renewal of stem cells [21–24, 42]. Several cytokines in the tumor microenvironment can activate the Wnt/β-catenin signaling pathway and thus increase the stemness of cancer cells [43, 44]. Our study further demonstrated that greater matrix stiffness induced Wnt/β-catenin signaling by upregulating BCL9L, ultimately increasing the stemness of glioma cells. Based on our findings, we speculated that inhibiting Wnt/β-catenin could suppress glioma progression. Gigantol has been reported to suppress the growth of several tumor types [45], and we found that this Wnt/β-catenin inhibitor significantly improved the anticancer effects of traditional clinical interventions such as chemotherapy and radiotherapy. Thus, gigantol could be an effective adjuvant therapeutic for glioma treatment.

In summary, our study revealed the correlation between matrix stiffness and glioma development by demonstrating that a stiffer matrix enhanced the stemness of glioma cells, resulting in sustained tumor growth. Our investigation of the underlying mechanism indicated that higher matrix stiffness increased glioma stemness by activating the BCL9L/Wnt/β-catenin signaling pathway. The application of gigantol, a Wnt/β-catenin inhibitor, significantly improved the effectiveness of traditional clinical interventions, implying that gigantol could be a feasible adjuvant agent for clinical glioma treatment. Our results also suggested that the matrix stiffness and BCL9L levels of glioma tissues could be potential biomarkers for glioma diagnosis or tumor progression analysis.

## MATERIALS AND METHODS

### Cell lines and reagents

The human glioma cell lines LN229 and T98G were purchased from the Cell Bank of the Chinese Academy of Sciences (China). LN229 and T98G cells were cultured in Dulbecco’s modified Eagle’s medium (Gibco, USA) supplemented with 10% fetal bovine serum (Gibco, USA) at 37° C. Gigantol and KYA1797K were purchased from MCM (USA). Temozolomide was purchased from Sangon (China). All other chemical reagents were of high-performance liquid chromatography grade and purchased from Solarbio (China).

### Patients and specimens

Human glioma tumor tissue samples were obtained from the Affiliated Hospital of Southwest Medical University. The glioma patients did not receive chemotherapy or radiotherapy before the surgery. The protocols were approved by the ethics board of the Affiliated Hospital of Southwest Medical University. Written informed consent was obtained from all subjects, and all methods were performed in accordance with the Declaration of Helsinki.

### Preparation of polyacrylamide gels of different stiffness levels

Polyacrylamide gels of different stiffness levels were used for cell culture, as reported previously [39]. Briefly, coverslips were coated with a thin layer of gel containing a mixture of 3-10% acrylamide and 0.01%-0.3% bis-acrylamide, resulting in gels with stiffness levels of 2, 8 and 16 kPa. Polyacrylamide gel polymerization was promoted by the addition of 10% ammonium persulfate (1/100 volume) and tetramethylethylenediamine (3/1000 volume). The coverslips were washed twice for 20 minutes each in PBS on a rocker, and then were sterilized in a PBS solution for one hour with ultraviolet light. Subsequently, 50 μL of heterobifunctional sulfosuccinimidyl 6-(4′-azido-2′-nitrophe-nylamino) hexanoate was added and photoactivated for five minutes with ultraviolet light. After being rinsed with a PBS solution, the coverslips were coated with 10 μL/mL fibronectin for 1.5 hours and rinsed before cell seeding. The gels were soaked in serum-free culture medium for 24 hours before usage. All elements of the polyacrylamide gels were purchased from Solarbio (China).

### Atomic force microscopy analysis

For sample preparation, all steps were performed in a sterile PBS solution supplemented with phenylmethylsulfonyl fluoride (Beyotime, China). Each biological sample was immobilized on a 35-mm plastic plate with 502 super glue (Deli, China). After treatment, the samples were washed twice with a PBS solution and imaged under a BioScope Resolve atomic force microscope (Bruker, USA). Tissue stiffness was measured on the microscope as reported previously [46].

### Gene interference

For *BCL9L* silencing in tumor cells, T98G and LN229 cells were infected with a *BCL9L* shRNA lentivirus (Hanbio, China) according to the manufacturer’s protocol. The *BCL9L* shRNA sequences were 5′-CCGGGCAATGTTGCAAATACGATAACTCGAGTTATCGTATTTGCAACATTGCTTTTTTG-3′ (shRNA #1) and 5′- CCGGGCCTAGCAACTCAAGTCTGAACTCGAGTTCAGACTTGAGTTGCTAGGCTTTTTG-3′ (shRNA #2).

For *BCL9L* overexpression, cDNAs for human BCL9L were synthesized by Sino Biological Inc, China, The BCL9L cDNAs were inserted into pLVX-EF1α-IRES-Puro -lentiviral vector (Takara, Japan) with an C-terminal 3×Flag tag for stable expression in T98G and LN229 cells.

### Cell proliferation and colony formation assays

For the cell proliferation analysis, human glioma cells were plated on a 96-well plate (5000 cells/well). Cell proliferation was measured every 24 hours, and the absorbance was read at 450 nm. For the colony formation assay, LN229 or T98G cells (~500 cells/well) were seeded in a six-well plate. After 14 days, the cell colonies were imaged and counted.

### Western blotting

Radioimmunoprecipitation assay buffer (Beyotime, China) containing protease inhibitors (Beyotime, China) was used to lyse the treated cells, and total proteins were extracted at 4° C. Then, 60-μg protein samples were separated via sodium dodecyl sulfate polyacrylamide gel electrophoresis and transferred onto nitrocellulose membranes. The following primary antibodies were used: anti-BCL9L (1:1000, ab113110, Abcam, UK), anti-β-actin (1:1000, ab6276, Abcam) and anti-β-catenin (1:1000, ab32572, Abcam). Proteins were detected using a chemiluminescence kit (Beyotime, China). The expression of β-actin was used as an internal control.

### Immunofluorescence staining

Glioma tissues dissected from patients were fixed in 4% paraformaldehyde for 96 hours and sectioned at a thickness of 4 μm. The sections were then deparaffinized and rehydrated in alcohol and water. Antigen retrieval was performed in sodium citrate buffer for five minutes at 100° C. Hydrogen peroxide (0.3%) was used to block peroxidase. Then, the sections were incubated with anti-BCL9L (1:1000, ab113110, Abcam) or anti-β-catenin (1:1000, ab32572, Abcam) primary antibodies at 4° C overnight. After being washed with PBS, the samples were incubated with goat anti-rabbit secondary antibodies (1:3000), and the nuclei were stained with 4′,6-diamidino-2-phenylindole (Solarbio, China). Images were obtained using a laser scanning confocal microscope (Leica, Germany).

### Flow cytometry

LN229 or T98G cells were cultured under different stiffness conditions for 48 hours. Then, the cells were harvested, washed twice with cold PBS buffer and stained with a CD133-APC antibody (eBioscience, USA) for 30 minutes at room temperature. The samples were then washed with PBS and analyzed on a BD-C6 flow cytometer (Becton Dickinson, USA).

### Animal protocols

All the animal care and experimental procedures were approved by the Animal Care and Use Committee of the Affiliated Hospital of Southwest Medical University. The experiments were performed in accordance with the Committee Guidelines on the Use of Live Animals in Research, which are based on the National Institutes of Health Guide for the Care and Use of Laboratory Animals. Female NSG mice (four to six weeks old) were purchased from Huafukang Co. (China) and raised under specific pathogen-free conditions.

For tumor growth analysis, LN229 or T98G cells (1×10^6^ cells in 100 μL of PBS) were subcutaneously injected into the right side of each NSG mouse. The mice were then randomly assigned to the various treatment groups (n=6 per group).

For tumorigenesis analysis, four-week-old female NSG mice were subcutaneously injected with LN229, U87 or T98G cells (1×10^4^) on the right side. After two weeks, the tumor number in each mouse was recorded.

For the analysis of anticancer therapeutic effects, LN229 or T98G cells (1×10^6^ cells in 100 μL of PBS) were subcutaneously injected into the right side of each NSG mouse. After 10 days, the mice were treated with PBS, chemotherapy, radiotherapy or chemotherapy/radiotherapy combined with gigantol once per week. After two weeks of treatment, the tumor sizes and survival times of the mice were recorded. Tumor size was measured using digital calipers, and the tumor xenograft volume was calculated as follows: length × width^2^ × 0.5.

### Statistical analysis

Each experiment was performed in triplicate. Data are expressed as the mean ± standard deviation of at least three independent experiments. Differences among groups were analyzed via analysis of variance or Student’s t-test using GraphPad Software (La Jolla, CA, USA) and SPSS 23.0 software (Chicago, IL, USA). Survival times were analyzed with a log-rank test. P < 0.05 was defined as a statistically significant difference.

## Supplementary Material

Supplementary Figures
