# Supplementary material for: Matrix stiffness promotes glioma cell stemness by activating BCL9L/Wnt/β-catenin signaling
Source: Aging (Albany NY). 2021 Feb 1;13(4):5284–96. doi: 10.18632/aging.202449 (PMC7950305; doi:10.18632/aging.202449)
Supplement: Supplementary Figures [file aging-13-202449-s001.pdf]

SUPPLEMENTARY FIGURES

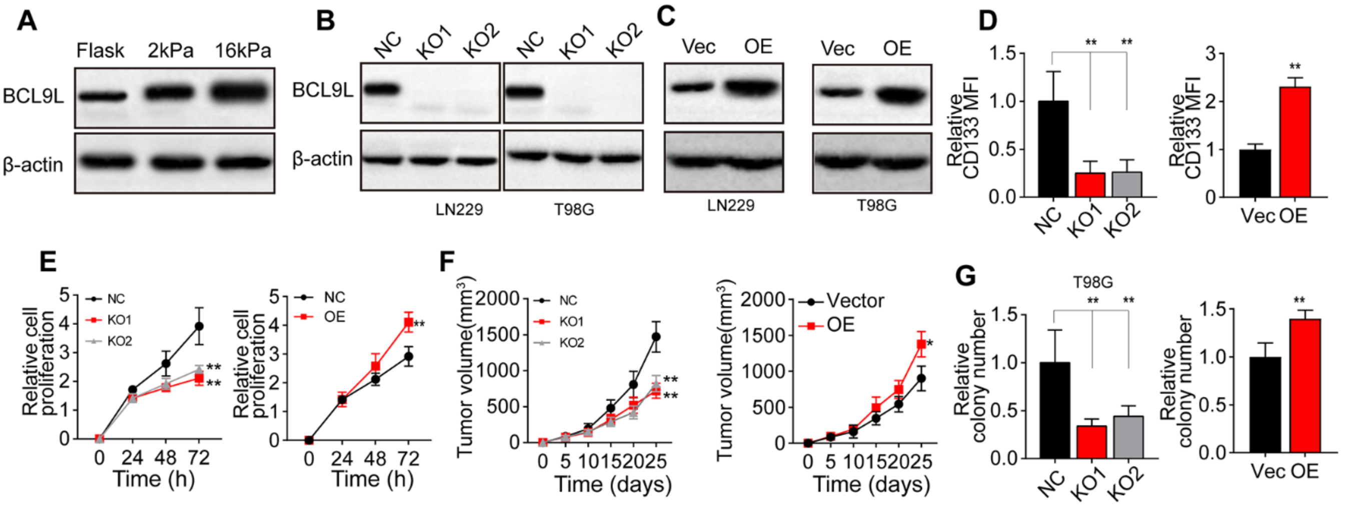

Supplementary Figure 1. Matrix stiffness regulated glioma stemness through BCL9L. (**A**) Western blotting analysis of BCL9L expression in T98G cell lines cultured on different stiffness gel. Western blotting analysis of BCL9L expression in LN229 or T98G cell lines transfected with (**B**) shBCL9L-RNA or (**C**) transfected with pCMV-BCL9L. (**D**)Flow cytometry analysis of CD133 expression in T98G-NC, T98G-KO1, T98G-KO2 cell lines cultured on 16Kpa stiffness gel or LN229-Vec and LN229-BCL9LOE cell lines cultured on flask dishes. (**E**) The cell proliferation of T98G-NC, T98G-KO1, T98G-KO2 cell lines pre-cultured on 16Kpa stiffness gel or LN229-Vec and LN229-BCL9LOE cell lines cultured on flask dishes. (**F**) Tumor volume was measured at various time points of T98G-NC, T98G-KO1, T98G-KO2 cell lines pre-cultured on 16Kpa stiffness gel or LN229-Vec and LN229-BCL9LOE cell lines cultured on flask dishes bearing mice. (**G**) The cell colonies formed assay of T98G-NC, T98G-KO1, T98G-KO2 cell lines pre-cultured on 16Kpa stiffness gel or LN229-Vec and LN229-BCL9LOE cell lines cultured on flask dishes was detected at various time points. *P < 0.05, **P < 0.01, n.s no significant difference.

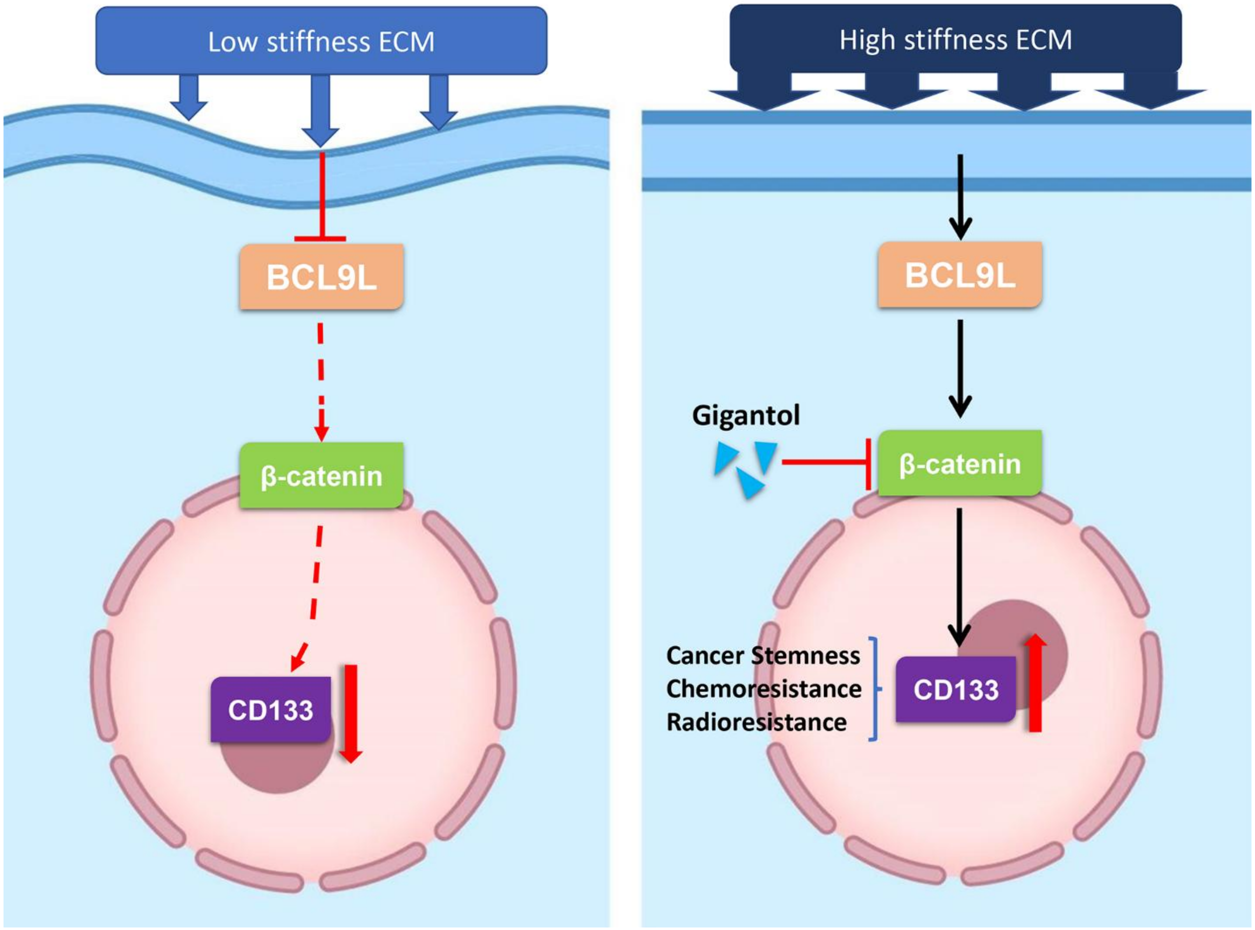

Supplementary Figure 2. Model for matrix stiffness promotes glioma cells stemness through a BCL9L/Wnt/β-catenin signaling pathway.
